# Biomimetic chitosan with biocomposite nanomaterials for bone tissue repair and regeneration

**DOI:** 10.3762/bjnano.13.92

**Published:** 2022-09-29

**Authors:** Se-Kwon Kim, Sesha Subramanian Murugan, Pandurang Appana Dalavi, Sebanti Gupta, Sukumaran Anil, Gi Hun Seong, Jayachandran Venkatesan

**Affiliations:** 1 Department of Marine Science and Convergence Engineering, College of Science and Technology, Hanyang University, Gyeonggi-do 11558, Korea; 2 Biomaterials Research Laboratory, Yenepoya Research Centre, Yenepoya (Deemed to be University), Deralakatte, Mangalore, Karnataka 575018, India; 3 Department of Dentistry, Oral Health Institute, Hamad Medical Corporation, College of Dental Medicine, Qatar University, Doha, Qatar; 4 Department of Bionano Engineering, Center for Bionano Intelligence Education and Research, Hanyang University, Ansan 426-791, South Korea

**Keywords:** antibacterial activity, biomimetic materials, bone graft substitutes, chitosan, gold, osteoinductive, silver

## Abstract

Biomimetic materials for better bone graft substitutes are a thrust area of research among researchers and clinicians. Autografts, allografts, and synthetic grafts are often utilized to repair and regenerate bone defects. Autografts are still considered the gold-standard method/material to treat bone-related issues with satisfactory outcomes. It is important that the material used for bone tissue repair is simultaneously osteoconductive, osteoinductive, and osteogenic. To overcome this problem, researchers have tried several ways to develop different materials using chitosan-based nanocomposites of silver, copper, gold, zinc oxide, titanium oxide, carbon nanotubes, graphene oxide, and biosilica. The combination of materials helps in the expression of ideal bone formation genes of alkaline phosphatase, bone morphogenic protein, runt-related transcription factor-2, bone sialoprotein, and osteocalcin. In vitro and in vivo studies highlight the scientific findings of antibacterial activity, tissue integration, stiffness, mechanical strength, and degradation behaviour of composite materials for tissue engineering applications.

## Introduction

Bone-related defects and diseases are a serious concern to the life of patients [1]. Autografts, allografts, and synthetic grafts are frequently utilized by clinicians to treat bone defects. Bone grafts should have osteoconductive, osteoinductive, and osteogenic properties to mimic the natural function of the bone [2]. Autografts are considered the gold-standard bone graft substitute since it has all three properties previously mentioned. Hence, the success rate of autograft techniques is always maximum. On the other hand, allograft and synthetic grafts are good in terms of osteoconductive properties; however, osteoinductive and osteogenic properties are limited. Cells and growth factors provide the osteoinductive and osteoconductive properties to bone graft substitutes. Therefore, to get all the three properties of bone graft substitutes, synthetic biomaterials are often mixed with growth factors and host-derived cells to increase bone formation [3]. Bone tissue engineering is the process of developing bone graft biomaterials with the combination of materials, growth factors, and cells [4]. Both natural and synthetic materials have been extensively studied for bone graft substitutes [5–6]. Natural biomaterials often are biocompatible, biodegradable, and less toxic to cells, whereas synthetic biomaterials have excellent mechanical strength, uniform raw materials quality, and are abundant. Chitosan is a natural polymeric substance and an extensively studied material for bone tissue engineering due to its biocompatibility, biodegradability, and antimicrobial properties [7–10]. Chitosan in combination with silver, gold, copper, titanium oxide, zinc oxide, carbon nanotubes, graphene oxide, and biosilica was developed to improve bone scaffolds for better bone tissue repair and regeneration [11]. In tissue engineering applications, nanoscale topological characteristics influence cell adhesion, survival, proliferation, and differentiation. The rough surface of the materials at the nanoscale helps cellular peptide adhesion for better stem cell growth and differentiation [12–13]. Nanomaterials have several advantages such as high surface area, increased mechanical strength, and induction of several important genes for bone tissue repair and regeneration [14]. Nanomaterials such as silver [15], gold [16–17], titanium oxide [18], zinc oxide [19–20], carbon nanotubes [21–22], graphene [23] and biosilica have been studied in terms of their osteogenic potential in stem cell differentiation. Chitosan materials are often combined with these nanomaterials to fabricate a scaffold that can potentially mimic the natural structure and function of the bones. The addition of nanomaterials to chitosan provides several advantages including the retaining of biological activities of nanomaterials and prevention of particle aggregation [24–25].

## Review

### Bone structure

**Figure 1:**
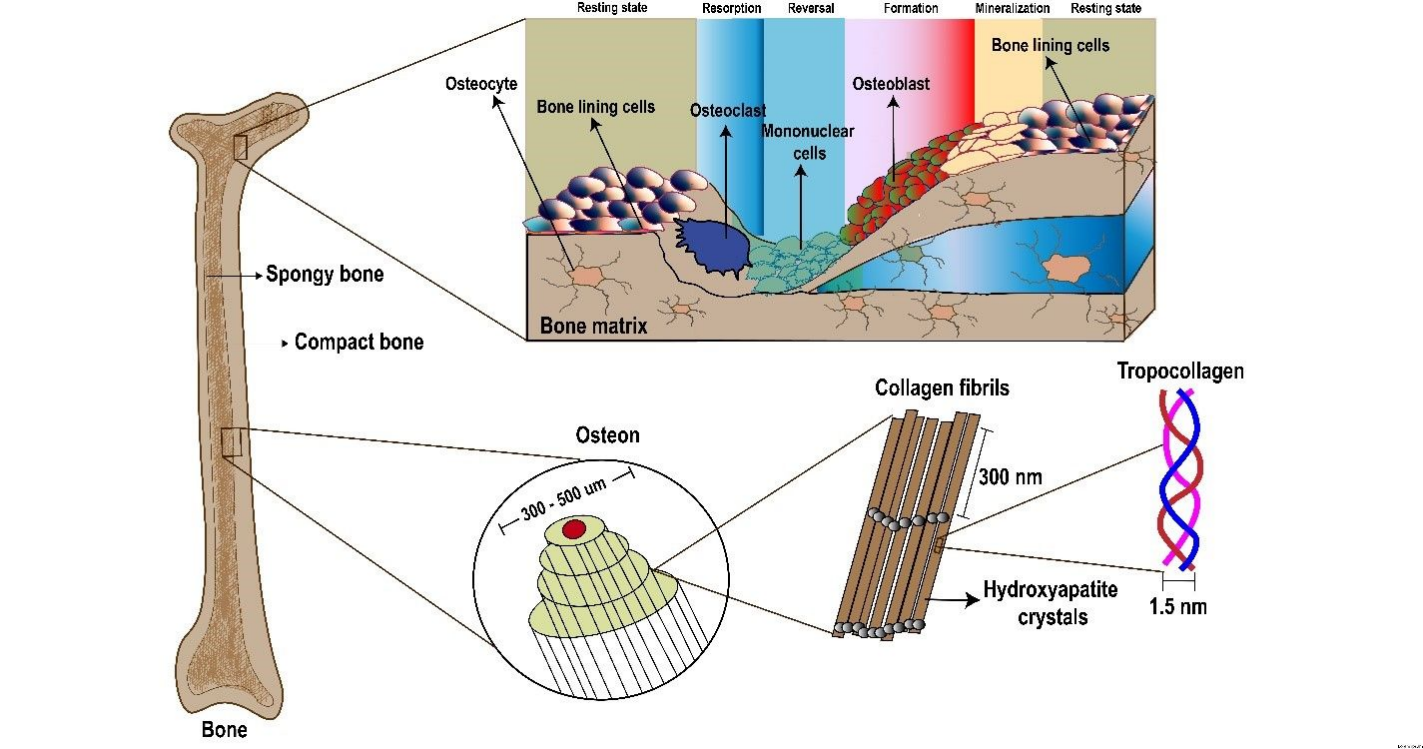
The bone structure. The magnified image shows the physiological arrangement of the bone matrix. The internal structure of the bone has the 100–500 µm osteon which contains 300 nm collagen fibrils, hydroxyapatite crystals, and 1.5 nm tropocollagen.

Bone is a hard tissue that contains different kinds of cells including osteoblasts, osteocytes, and osteoclasts (Figure 1) [26]. The inorganic hydroxyapatite and organic type I collagen components are vital to bone tissue. The bone biomineralisation activities are formed by nanosized hydroxyapatite crystals with connective collagen fibrils [27]. The bone possesses a unique combination of strength and stiffness, and it has excellent compressive strength and tensile strength due to the attribution of deep nanostructures of inorganic and organic components.

Human bones are complex and their asymmetric matrix is constituted of basic components hierarchically organized into distinct structural layers at macro- and nanoscale levels. Cortical (compact) and cancellous (trabecular) bones are two kinds of bone classification based on their macrostructure. A femur is a long bone with a thick cortical covering which is porous and has a cancellous interior. The calvaria is a flat bone with cortical layers on the outside and a cancellous structure on the inside [28–29]. The physical behaviour of the cortical bone is mainly controlled by porosity, mineralization rate, and solid matrix structure (cancellous interior) [30]. Also, the mechanical properties of cancellous bones are controlled by the structural organization of the matrix [31]. The bone microstructure mainly comprises collagen threads of lamellae coiled around layers to form a 200–250 µm diameter osteon which can vary between cortical and cancellous bones [31]. At the scale of 1 µm, collagen fibrils are surrounded by minerals [32] (Figure 1). Crystals, collagens, and non-collagen organic proteins are found at sub-nanoscale levels ranging from 1 to 10 nm [33]. It has been reported that 90% of the proteins identified inside the bone extracellular matrix is produced by bone-forming osteoblasts with a repeating amino acid sequence of [Gly(glycine)–X–Y]*_n_*, where X and Y may be proline and hydroxyproline. Collagen fibrils, composed of specific proteins, are usually responsible for mechanical strength. Furthermore, osteoblasts generate a membrane that includes alkaline phosphatase, which cleaves phosphatase groups and causes calcium and phosphate precipitation, resulting in the formation of natural bone minerals with a ratio of 1.67 [27].

Osteoblasts have been predominantly derived from mesenchymal stem cells, which express particular genes for the production of bone morphogenic proteins and wingless (Wnt) pathway elements. It has been revealed that runt-related transcription factors-2 (Runx2), osterix (Osx), and the distal-less homeobox 5 (Dlx5) are primarily responsible for osteoblast differentiation. Specifically, the gene RUNX2 upregulates the genes for collagen type I alpha 1 (ColIA1), alkaline phosphatase (ALP), bone sialoprotein (BSP), bone gamma-carboxyglutamate protein (BGLAP), and osteocalcin (OCN), which are important for the regeneration process [34]. To mimic the natural bone function, the composite materials should be in the form of inorganic and organic composites. To mimic the inorganic portion, researchers have tried to utilize calcium phosphate materials due to their similarity to the native tissue. To mimic the organic portion of the bone, several materials including polymers, proteins, and carbon-based materials have been tested.

### Biomaterials for bone graft substitutes

Hydroxyapatite and its composites have been widely utilized/studied biomaterials for bone tissue engineering [35–36]. Hydroxyapatite with several polymeric materials has been used to mimic the natural function of the bone. Different kinds of polymeric materials have been utilized including chitosan, alginate, fucoidan, carrageenan, and ulvan from natural polymeric materials. Polycaprolactone (PCL), poly ᴅ,ʟ-lactic-*co*-glycolic acid (PLGA), and polylactic acid (PLA) have been extensively studied with hydroxyapatite to develop bone mimetic scaffolds [37]. Bone morphogenetic protein 2 (BMP-2) is one of the widely utilized growth factors for the treatment of bone-related diseases and defects [38–39]. Growth factors are responsible for bone formation which happens through the stimulation of different kinds of cells in our body. However, the drawbacks of using growth factors and enzymes are stability, high cost, and low availability. To overcome these issues, bioactive materials are often studied to mimic the natural function of growth factors. Several researchers are studying nanomaterial-based chitosan composites regarding their osteoinductive properties [40–41]. Chitosan is combined with several polymeric materials and nanoparticles to mimic the natural function of the bone (Table 1) [42–43]. Chitosan biomaterials enhance the proliferation of osteoblasts and the formation of bone minerals by promoting gene expression of type I collagen, osteopontin, osteonectin, and osteocalcin. Chitosan mixed with different functionalized materials such as silver, magnesium oxide, and bioactive glass has aided the treatment of infected bone defects with a biodegradable behaviour at the bone defect site [44–45]. The *N*-(2-hydroxy)propyl-3-trimethylammonium chitosan chloride (HACC) is a chitosan biomaterial used for the treatment of infected bone defects. The arrangements of amino acids in the chitosan (degree of deacetylation) determines the growth and support necessary for osteoblast differentiation. Increasing the degree of deacetylation in the chitosan, the positive charge density increases, which results in high electrostatic interaction which electrically stimulate the osteoblasts to proliferative and differentiate [46–48]. Chitosan biomaterials containing graphene oxides were used as substrates for the generation of hydroxyapatite, which has a high elastic flexibility and tensile strength for bone tissue engineering applications [49]. Carbon nanotubes were also used as fillers in chitosan to increase flexibility, porosity, and mechanical strength of chitosan biomaterials for applications in bone tissue engineering [50]. The porous structure of the chitosan with an absorbable collagen sponge encourages osteoblast stem cells to attach to the surface to proliferate and differentiate promoting bone development. As compared to absorbable collagen sponges, the increase in bone mineral density, defect closure, and new bone formation on rat calvaria defects indicate a strong healing effect and new bone formation on chitosan/absorbable collagen sponges [51].

**Table 1:** Combinations of chitosan with several polymeric materials and nanoparticles to mimic the natural bone function.

S. No.	Materials	Methods	Cell line/animal	Bacteria	Ref.

1	chitosan–silver	coating	rabbit	*Staphylococcus aureus*	[52]
2	chitosan–silver	electrophoretic deposition	MG-63 cells/rat	*Staphylococcus aureus*	[53]
3	chitosan–diatomite	freeze-drying	MG-63 cells/Saos-2 cells/human osteoblasts	–	[54]
4	chitosan–silica	cross-linking	–	–	[55]
5	chitosan–silver	self-assembly	–	*Staphylococcus aureus* and *Escherichia coli*	[56]
6	chitosan–collagen	cross-linking	MC3T3-E1 cells	–	[57]
7	chitosan–carbon nanotubes	sonication	–	–	[58]
8	chitosan–carbon nanotubes	electrophoretic deposition	MC3T3-E1 cells	–	[59]
9	chitosan–reduced graphene oxide	self-assembly	MG-63 cells	–	[60]
10	chitosan–graphene oxide	sonication and lyophilisation	MC3T3-E1 cells	–	[61]
11	chitosan–graphene oxide	solvent casting	MG-63 cells	*Staphylococcus aureus* and *Staphylococcus epidermidis*	[62]
12	chitosan–tetraethoxysilane	sol–gel	human osteoblasts	–	[63]
13	chitosan–bioactive glass	sol–gel/coprecipitation	human osteosarcoma cells	–	[64]
14	chitosan–mesoporous silica nanoparticles	electrospinning	MC3T3-E1 cells	–	[65]
15	chitosan film–graphene oxide–hydroxyapatite–gold	hydrothermal and gel casting	C3H10T1/2	*Escherichia coli*, *Streptococcus mutans*, *Staphylococcus aureus*, and *Pseudomonas aeruginosa*	[66]
16	chitosan–silver nanoparticle	reduction	human adipose-derived mesenchymal stem cells	–	[67]
17	chitosan–carbon nanotubes–gelatin	sonication	–	*Bacillus subtilis*, *Staphylococcus aureus* and *Listeria monocytogenes*, *Escherichia coli* 0157, *Salmonella enteritidis*, *Salmonella typhi*, and *Klebsiella pneumoniae*	[68]
18	chitosan–hydroxyapatite–zinc oxide	stirring and self-assembly	MG-63 cells	*Escherichia coli* XL1B, *Lysinibacillus fusiformis*, and *Bacillus cereus*	[69]
19	chitosan–hydroxyapatite–zinc oxide–palladium	coating	dental pulp stem cells	*Pseudomonas aeruginosa*	[70]
20	chitosan–zinc–gelatin	electrophoretic deposition	rat bone marrow stromal cells/Sprague Dawley rat	*Escherichia coli* and *Staphylococcus aureus*	[29]
21	chitosan–graphene oxide–hydroxyapatite	ultrasonication	MG-63 cells	–	[71]
22	chitosan–graphene oxide–polyvinylpyrrolidone	electrospinning	rat bone marrow mesenchymal stem cells/Sprague Dawley rat	–	[72]
23	chitosan–graphene oxide–hydroxyapatite	layer-by-layer assembly technique	mouse mesenchymal stem cells	–	[73]
24	polysaccharide 1-deoxylactit-1-yl chitosan–silver nanoparticles	coating	human adipose-derived stem cells/mini-pig	*Staphylococcus aureus* and *Pseudomonas aeruginose*	[74]
25	chitosan–nanohydroxy- apatite–nanosilver	freeze-drying	osteoprogenitor cells	*Staphylococcus aureus* and *Escherichia coli*	[75]
26	chitosan–polyurethane–silver nanoparticle	electrospinning	NIH 3T3 cells	*Porphyromonas gingivalis*	[76]
27	carboxylated chitosan–silver–hydroxyapatite	facile gas diffusion	MG-63 cells	*Staphylococcus aureus*	[77]
28	chitosan–nanohydroxy- apatite–silver	cross-linking and lyophilisation	MC3T3-E1 cells	*Escherichia coli*	[78]
29	chitosan–nanohydroxy- apatite–silver	in situ hybridization	human osteoblasts	*Escherichia coli*	[79]
30	chitosan–multiwalled carbon nanotubes–hydroxyapatite	sonication and cross-linking	MC3T3-E1 cells	–	[80]
31	titanium oxide–naringin–chitosan	dropping	osteoblasts	–	[81]
32	chitosan–nanosilicon dioxide–chondroitin sulfate	cross-linking	MG-63 cells	–	[82]
33	chitosan–nanosilicon dioxide-gelatin	cross-linking	MG-63 cells	–	[83]
34	chitosan–octa(tetramethyl- ammonium)–polyhedral silsesquioxane	Freeze-drying	MG-63 cells/ Saos-2 cells/3T3 cells	–	[84]
35	chitosan–bioactive glass–silver nanoparticle	electrophoretic deposition	MG-63 cells	*Staphylococcus aureus*	[85]
36	carboxymethyl chitosan–copper ion–alginate	cross-linking	MC3T3-E1 cells	*Staphylococcus aureus*	[86]
37	chitosan–glycyl-ʟ-histidyl-ʟ- lysine–copper ions–mesoporous silica nanoparticles	stirring	MC3T3-E1 cells	*Escherichia coli* and *Staphylococcus aureus*	[87]
38	chitosan–nanohydroxy- apatite–silver–magnesium ions	microwave-assisted coprecipitation	human fibroblast skin cells	*Escherichia coli* and *Staphylococcus aureus*	[88]
39	chitosan–titanium oxide–selenium	electrodeposition	osteoblasts	*Escherichia coli*	[89]
40	chitosan–silver–ion-loaded calcium phosphate	electrospinning	bone marrow stromal cells	*Staphylococcus mutans*	[90]
41	chitosan–carboxymethyl cellulose–silver nanoparticle modified cellulose nanowhiskers	freeze-drying	MG-63 cells	*Escherichia coli* and *Enterococcus hirae*	[91]
42	chitosan–silver–strontium– hydroxyapatite	ultrasonication	human bone marrow mesenchymal stem cells	*Staphylococcus aureus*	[92]
43	chitosan–collagen–functional- ized multiwalled carbon nanotubes–hydroxyapatite	lyophilisation/freeze drying	MG-63 cells	–	[93]
44	chitosan–zein–polyurethane– functionalized multiwalled carbon nanotubes	electrospinning	MC3T3-E1 cells	*Escherichia coli*, *Staphylococcus aureus*, *Micrococcus luteus*, and *Staphylococcus epidermidis*	[94]
45	chitosan–titanium substrate–titanium oxide nanotubes–alginate	self-assembly	osteoblasts	*Escherichia coli* and *Staphylococcus aureus*	[95]
46	chitosan–melatonin–titanium oxide–gelatin	spin-assisted layer-by-layer	mesenchymal stem cells	–	[96]
47	chitosan–alginate–hydroxy- apatite–silver nanoparticles	freeze-drying	MG-63 cells	*Escherichia coli*, *Staphylococcus epidermidis*, *Staphylococcus aureus*, and *Pseudomonas aeruginosa*	[97]
48	chitosan–copper–bioactive nanoparticles–glycero- phosphate–silk fibroin	stirring	MC3T3-E1 cells/rat	–	[98]
49	chitosan–titanium–silica– silver–acemannan	induction plasma spray coating	osteoblasts/rat	*Staphylococcus epidermidis*	[99]
50	chitosan–nanohydroxyapatite– nanocopper–zinc	freeze-drying	rat progenitor cells	*Escherichia coli* and *Staphylococcus aureus*	[100]
51	chitosan–calcium phosphate–graphene oxide–silver nanoparticles	freeze-drying	bone marrow stromal cells/Sprague Dawley rat	*Staphylococcus epidermidis* and *Escherichia coli*	[101]
52	chitosan–silver-doped hydroxyapatite–iron oxide	ball milling	NIH-3T3 cells	*Staphylococcus aureus* and *Escherichia coli*	[102]
53	chitosan–bone morphogenic protein–silver–hydroxyapatite	coating	bone marrow stromal cells/Japanese big-ear white rabbit	*Staphylococcus epidermidis* and *Escherichia coli*	[103]
54	chitosan–silk cryogel–silver–strontium– nanohydroxyapatite	freeze-drying	rat bone marrow stromal cells/Sprague Dawley rat	*Escherichia coli* and *Staphylococcus epidermidis*	[104]
55	chitosan–calcium silicate–gelatin–silver	coating	MG-63 cells	*Staphylococcus aureus* and *Escherichia coli*	[105]
56	chitosan–silver–magnesium– strontium– iron–hydroxide	ultrasonication	human bone marrow mesenchymal stem cells	*Staphylococcus aureus*	[106]
57	chitosan–nanohydroxyapatite– gelatin–nanocopper–zinc	freeze-drying, cross-linking and lyophilisation	mouse embryonic fibroblasts/rabbit	–	[107]
58	chitosan–poly (3-hydroxybutyrate)–multiwalled carbon nanotubes–nano- bioglass–titania	coating	MG-63 cells	–	[108]

### Chitosan with metal nanomaterials for bone tissue engineering

#### Chitosan–silver nanocomposites

**Figure 2:**
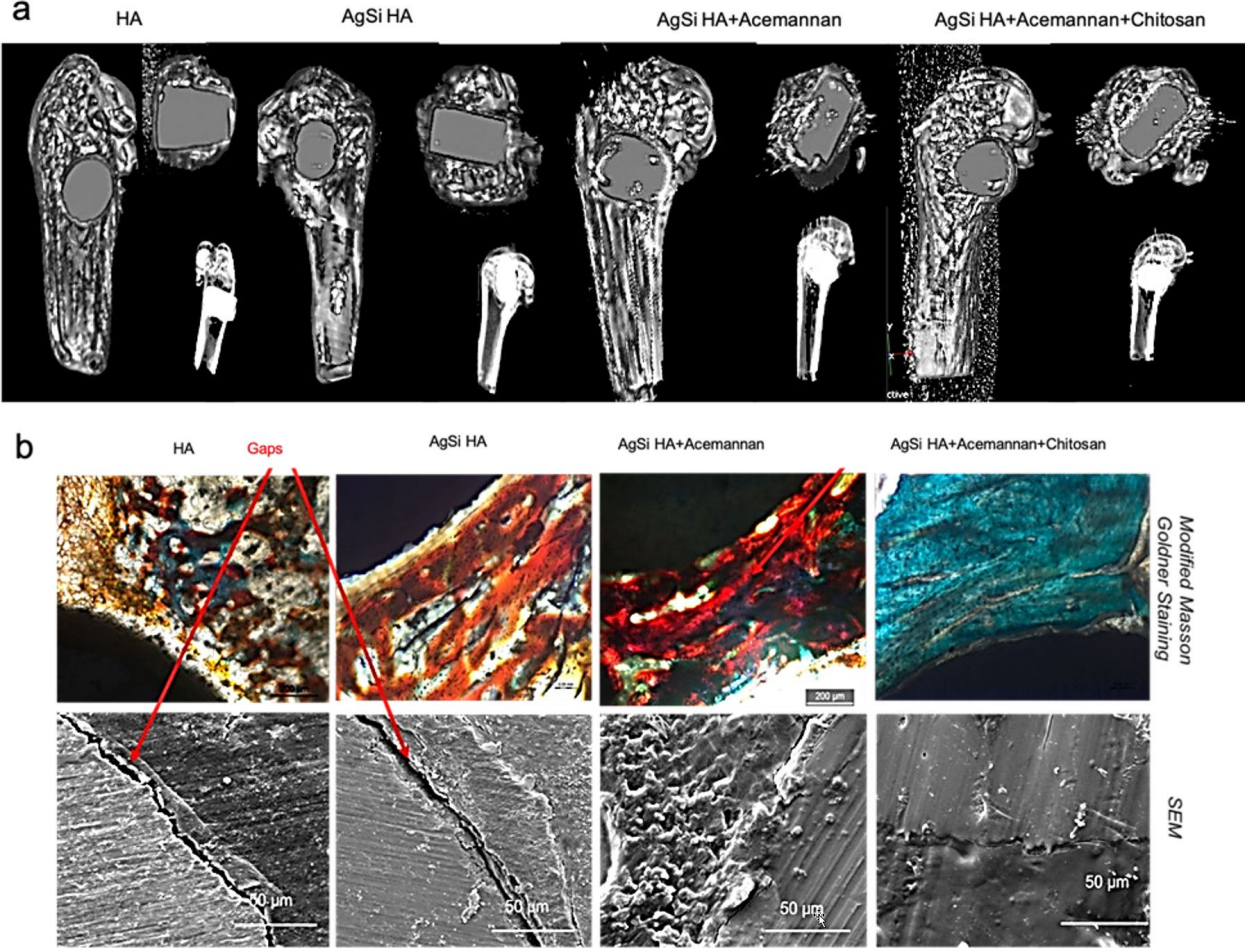
The in vivo development of new bone in a rat distal femur model five weeks after implantation. Figure 2a depicts 3D pictures created by computed tomography (CT) scan radiographs which show good bone lodging in all implants. There are noticeable holes at the hydroxyapatite implant interface. Ag–Si hydroxyapatite implants, on the other hand, have a smooth contact between the host bone and the implant. Acemannan and chitosan coatings have been detected. Figure 2b depicts an optical microscopy image of Masson-Goldner staining which shows that acemannan and chitosan accelerates the formation of new bone. Figure 2 was reprinted with permission from [99], Copyright 2019 American Chemical Society. This content is not subject to CC BY 4.0.

Silver nanoparticles (AgNPs) have gained much attention in bone-related implant research due to its intrinsic antimicrobial properties. Xie et al. (2019) developed AgNPs composites containing polydopamine–hydroxyapatite–chitosan by adding AgNPs to hybrid materials, which significantly reduced microbial infection in the implanted place [109]. Polydopamine and chitosan play an important role in the controlled release of AgNPs at the implanted site and also behave as biocompatible scaffolds. Wang et al. (2019) developed a system containing hydroxyapatite and silver-based composites and electrodeposited those onto titanium implants and chitosan to regulate silver ion and calcium ion release [110]. Silver and antibiotic drugs were mixed into the biocomposite containing chitosan, graphene oxide, and hydroxyapatite. Furthermore, using one-step electrodeposition, biocomposites were coated on titanium. It has been discovered that the addition of graphene oxide and chitosan improves mechanical strength and cell adhesion. Importantly, the developed biocomposites have excellent antimicrobial activity [111]. AgNP-loaded fibrillar collagen–chitosan matrix was used for further biomineralisation using a simulated body fluid (SBF) solution. The developed composites show better mineralisation and also significant antimicrobial activity [112]. The hydroxyapatite was coated onto titanium by using an induction plasma-spray coating approach. The hydroxyapatite was then buffed with silver and silica. In the SBF solution, the release of acemannan from the coating material was also evaluated. Acemannan was found to be released in a long-term manner. Furthermore, an in vitro cell interaction study on osteoblasts revealed that the produced material stimulates cell growth. In addition, in vivo investigations on a rat distal femur model show that a considerable amount of new bone has grown (Figure 2). Besides this, significant antibacterial properties against *Staphylococcus epidermidis* have been found [99]. Coating materials containing chitosan, bioactive glass, and AgNPs were developed by using the electrophoretic deposition method. The produced material was coated on stainless steel 316 substrates. Further, the developed material shows apatite formation in SBF and it stimulates the growth of MG-63 osteoblast-like cells. In addition, antibacterial activity was discovered against *Staphylococcus aureus* [85].

#### Chitosan–gold nanocomposites

Gold nanoparticles (AuNPs) have been extensively studied for tissue engineering, drug delivery, diagnostic, and bioimaging applications due to its biocompatibility, tuneable size, and ability to cover different kinds of therapeutic agents. Gold nanoparticles control osteoclastic differentiation [113–115]. Osteogenic properties of AuNPs were proven by in vitro cell interaction with mesenchymal stem cells through the p38 MAPK signalling pathway [17]. Gold nanoparticles show promising results in bone marrow mesenchymal stem cell differentiation towards osteogenic lineages, which might be due to the size and intrinsic factors of AuNPs. Mahmoud et al. (2020) have carried out a study on bone regeneration efficiency by using the AuNPs, hydroxyapatite nanoparticles, chitosan nanoparticles, gold hydroxyapatite-based nanocomposites, and chitosan–hydroxyapatite-based nanocomposites. In vitro cell interaction with bone-marrow-derived mesenchymal stem cells was used to assess the osteogenic differentiation potential of these nanoparticles. Alizarin red S test also confirms the development of mineralized nodules. Furthermore, qRT-PCR analysis reveals that runt-related transcription factor-2 and bone morphogenic protein-2 gene expression was upregulated*.* The in vitro biological assays prove that AuNPs possess excellent osteoinductive properties [116]. In another research study, Choi et al. (2015) developed chitosan-mobilized AuNPs for treating bone defects. Chitosan was employed as a reducing agent to produce AuNPs. The developed AuNPs promote osteogenic differentiation in human adipose-derived mesenchymal stem cells. The proliferation of the cells was demonstrated by the alamarBlue test. Further, qRT-PCR analysis showed upregulation of alkaline phosphatase, bone sialoprotein, and osteocalcin gene expression. The Alizarin red S test was carried out by culturing human adipose-derived mesenchymal stem cells with chitosan-conjugated AuNPs, which shows calcium deposition confirming that developed nanoparticles promote osteogenic differentiation of human adipose-derived mesenchymal stem cells. The protein expression of human adipose-derived mesenchymal stem cells related to β-catenin signalling for the osteogenic process was confirmed by Western blot analysis [67].

#### Chitosan–copper nanocomposites

Several research discoveries have proven the osteogenic properties of copper nanoparticles (CuNPs). Injectable hydrogels comprising copper with bioactive nanoparticles/chitosan/glycerophosphate/silk fibroin were fabricated for bone regeneration applications. Further, in vitro biological assays performed with MC3T3-E1 cells prove the osteogenic properties of the fabricated hydrogel. In addition, in situ experiments were conducted on rat calvarial bone defects for eight weeks. The emergence of new bone in the defective region is confirmed by microscale computational micrographs and staining assay findings [98]. Ning et al. (2019) developed glycyl-ʟ-histidyl-ʟ-lysine-containing copper ions integrated with mesoporous silica nanoparticles (MSN) and chitosan. Further, the release of copper ions from the composite was investigated by soaking it into a buffer solution. In addition, biological assays were done with MC3T3-E1 cells. It has been found that the developed material is biocompatible and enhances osteogenic gene expression. Besides this, remarkable antibacterial properties against *Escherichia coli* and *Staphylococcus aureus* were obtained [87]. In another study, Lu et al. (2018) developed a scaffolding system of copper ions incorporated in carboxymethyl chitosan and alginate for bone tissue repair. The porous size of the scaffolds was in the range of 45 to 107 μm and with an average of 73.06 ± 21.13 μm. In vitro biological assays were carried out with MC3T3-E1 cells. Biological assays reveal that the developed scaffold promotes cell adhesion and proliferation. Also, enhancement in alkaline phosphatase, inducement of mineralisation, and upregulation of osteogenic gene expressions were observed. In vivo studies were done on Sprague Dawley rats. Microscale computational analysis and histological analysis show that new bone has formed. In addition, the developed scaffold demonstrated remarkable antibacterial efficiency against *Staphylococcus aureus* [86]. To achieve higher osteogenic properties, scaffolds composed of nanocopper–zinc integrated with nanohydroxyapatite, gelatin, and chitosan were developed by the freeze-drying method. The scaffolds were developed with a diameter of 8 mm and thickness of 2.5 mm. The porosity ranges from 97.8 to 99.5% with a pore size of 113 to 143 µm. In vitro cell interaction investigations with mouse embryonic fibroblasts demonstrate the osteogenic capabilities of the scaffolds [107]. In another study, Tripathi et al. (2012) investigated the osteogenic properties of the scaffolding system composed of nanocopper–zinc, chitosan, and nanohydroxyapatite. The osteogenic properties of the fabricated scaffold were confirmed by biological assays with rat osteoprogenitor cells. In addition, the scaffold shows excellent antibacterial activity against *Staphylococcus aureus* and *Escherichia coli* [100].

#### Chitosan–titanium oxide nanocomposites

Titanium and titanium-based alloy materials are often utilized in the biomedical field especially in bone tissue engineering due to several important properties which include excellent biocompatibility, higher resistance against corrosion, significant structural rigidity, and tolerance to body fluids [117–118]. A recent study has proven that synthetic changes made to the surface of titanium oxide (TiO_2_) nanotubes can influence their characteristics, substantially impacting cell contacts and antibacterial activity. As a result, antibiotic drugs combined with a surface-modified TiO_2_-based drug delivery system in synthetic bone implants can stimulate bone regeneration while preventing bacterial infection. Furthermore, coating materials containing TiO_2_ can help drug stabilisation producing a long-term drug release profile [119]. The electrochemical anodization process was used by Lai et al. (2018) to create TiO_2_ nanotubes. Naringin was also dropped directly into TiO_2_ nanotubes, which were then subsequently covered with chitosan layers. There was a long-term release of naringin from the system. In vitro biological tests on osteoblasts revealed that the cells were viable and alkaline phosphatase activity was increased. In addition, Alizarin red S staining tests detected the development of mineralized nodules [81]. On the Ti substrate, TiO_2_ nanotubes carrying a gentamicin drug mixture were deposited. Furthermore, a combination of alginate and chitosan was utilized to cover the TiO_2_–gentamicin composite using a self-assembly method. The osteoblasts obtained from newborn rat calvaria bones were subjected to biological tests. Cells were viable and proliferating with the produced composite material. In addition, there was an increase in alkaline phosphatase activity. Aside from that, exceptional antibacterial activity was observed against *Staphylococcus aureus* and *Escherichia coli* [95]. Melatonin-loaded TiO_2_ nanotubes were synthesized by Lai et al. (2017). Subsequently, using a spin-based layer-by-layer approach, chitosan and gelatin-based films were applied. Further, an in vitro cell interaction study using mesenchymal stem cells showed that the fabricated biocomposite has exceptional osteogenic potential [96]. In another research study, Chen et al. (2013) fabricated selenium-incorporated and chitosan-covered TiO_2_ nanotubes. Further investigation demonstrates the antibacterial, anticancer, and osteogenic properties of the produced biocomposite [89].

#### Chitosan–zinc oxide nanocomposites

**Figure 3:**
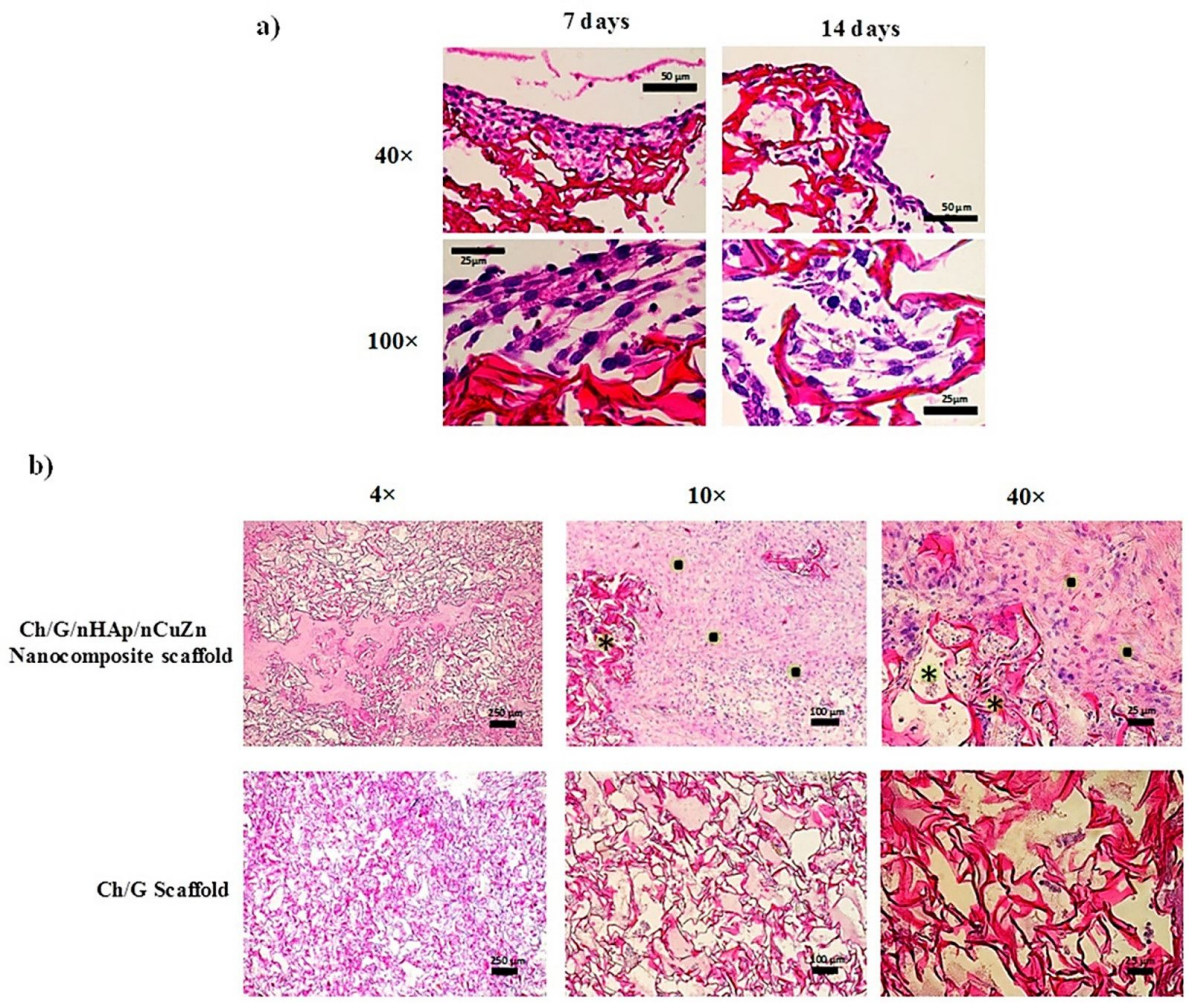
(a) Cross-sectional histological photomicrographs from the chitosan/gelatin/ nanohydroxyapatite/nanocopper/zinc alloy nanocomposite scaffold stained with haematoxylin/eosin at seven and 14 days after surgery. (b) Haematoxylin/eosin-stained sections of scaffolds from in vivo biocompatibility assessments. Figure 3 was reproduced from [107], (© 2017 J. C. Forero et al., published by MDPI, distributed under the terms of the Creative Commons Attribution 4.0 International License, https://creativecommons.org/licenses/by/4.0).

Zinc was utilized as a trace element in composites for bone tissue engineering applications since it improves bone density and minimizes bone loss. In this study, montmorillonite clay was modified with chitosan/hydroxyapatite–zinc oxide nanocomposites to mimic the natural bone matrix. The nanocomposites attain mechanical strength of 30.13 ± 0.16 MPa. This composite demonstrates antibacterial efficacy against Gram-negative and Gram-positive bacterial strains including *Escherichia coli*, *Lysinibacillus fusiformis*, and *Bacillus cereus*. The cytotoxicity behaviour was determined by an in vitro proliferation experiment with MG-63 cells. The findings indicate that the nontoxic substances are safe to be used in bone tissue engineering applications [69]. A new combination with palladium/zinc oxide/hydroxyapatite was fabricated and coated with different quantities of chitosan (0.125 and 0.25 g) to achieve higher compressive strength and toughness. The antibacterial activity of the composites was tested against Gram-negative bacteria (*Pseudomonas aeruginosa*) and the results show excellent activity and inhibition of bacterial growth at 25 µg/mL. The bioactivity of the composites was evaluated by simulation studies of body fluids. The results show that the composites can form hydroxyapatite bone minerals crystals in a ratio of Ca/P 1.67 [70]. Huang et al. (2017) have created a zinc-incorporated chitosan/gelatin nanocomposite to reduce surgical site infections. The nanocomposites were coated on titanium substrates and showed antibacterial efficacy against bacterial species of *Escherichia coli* and *Staphylococcus aureus*. The in vitro response of the nanocomposites was evaluated using rat bone marrow stromal cells. The results demonstrate that the composites had higher proliferation rate following the addition of the zinc trace element. Furthermore, the data indicate that cytotoxicity was reduced and also that the enzymatic alkaline phosphatase assay has significant activity [29]. The alkaline phosphatase assay was performed using mouse embryonic fibroblasts and the increased activity on the 21st day demonstrates a favourable environment for cell development and differentiation. The in vivo investigation was conducted on two male rabbits by making two 25 mm longitudinal incisions on the right and left dorsum. The bioimplants were placed and examined after four weeks of surgery. On the 14th day, histological studies revealed that cells had infiltrated into the holes. Meanwhile, degradation of the scaffold matrix was observed and represented as black spots in Figure 3 [107].

### Chitosan with carbon nanomaterials for bone tissue engineering

#### Chitosan–carbon nanotube composites

**Figure 4:**
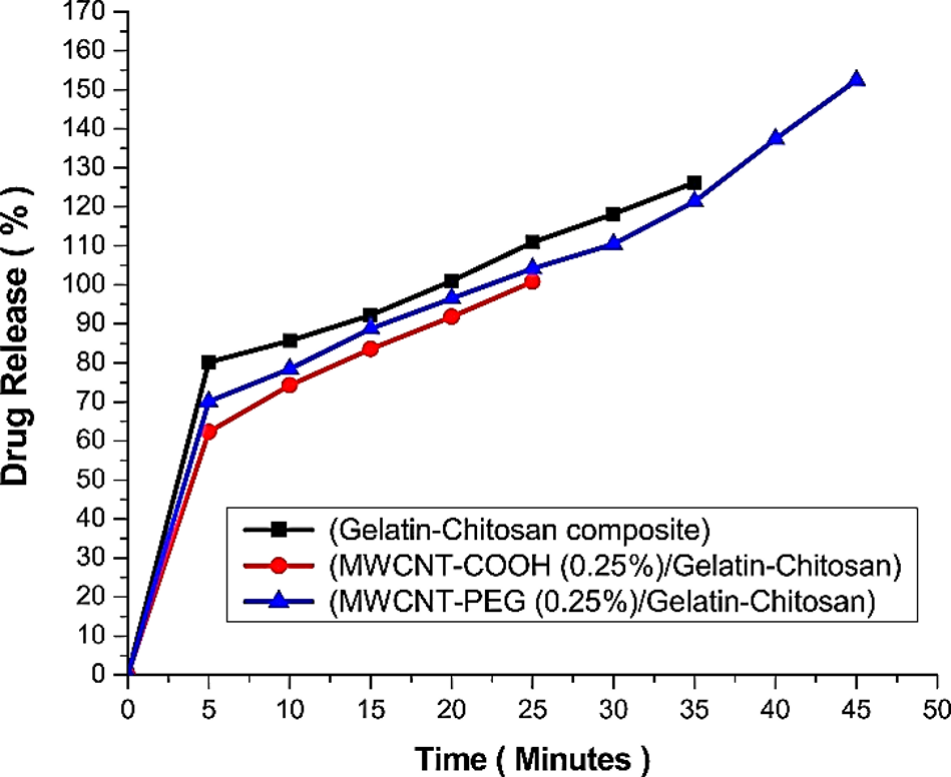
The ciprofloxacin release rate (%) from several composite films at pH 7.4. Figure 4 was reproduced from [68], (© 2018 S. Sharmeen et al., published by Elsevier, distributed under the terms of the Creative Commons Attribution-Non Commercial-No Derivatives 4.0 International License, https://creativecommons.org/licenses/by-nc-nd/4.0/). This content is not subject to CC BY 4.0.

**Figure 5:**
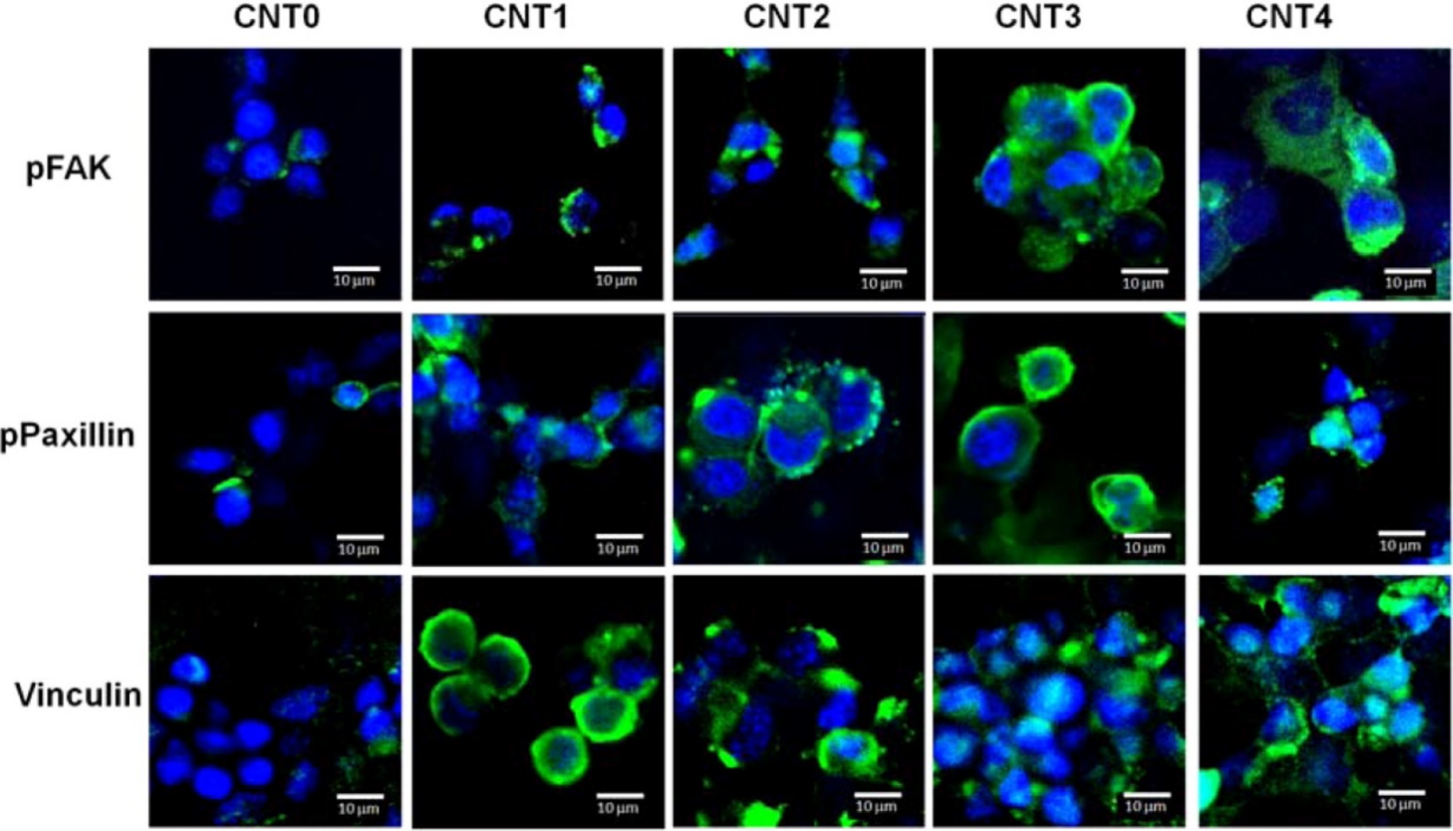
Adhesive molecules possibly involved in the initial cell adhesion events, analysed by immunostaining images. Signals positive for p-FAK, p-paxillin, and vinculin were strongly expressed in all the CNT–chitosan hybrid coatings but not in CNT0. Figure 5 was reprinted with permission from [59], Copyright 2014 American Chemical Society. This content is not subject to CC BY 4.0.

Carbon nanotubes have distinct physical, chemical, and optical properties that enable new bioengineering applications, notably in the development of natural bone tissue repair and replacement scaffolds. Carbon nanoparticles can provide a chemically and physically comparable microenvironment to that of the natural extracellular matrix, containing healing and stimulating components necessary for bone repair, making them a potential option for bone tissue regeneration. As carbon nanotubes are combined with natural polymers, such as chitosan and collagen, they develop an interlinked molecular framework with load-bearing applications and have superior mechanical qualities and biological advantages [57]. A further study reported that carbon nanotubes combined with synthetic polymers of poly-ʟ-lactide acid, polylactic acid, polyhydroxyethylmethacrylate, and polyethylene glycol were used in composite preparation for bone tissue engineering applications [10]. To create a fibrous scaffold for bone tissue regeneration, zein and chitosan were combined with polyurethane and functionalized multiwalled carbon nanotubes. The developed scaffolds have tensile strength of ≈7.05 MPa. According to the findings in vitro, the combination of scaffolds promotes fast cell-to-cell contact, which boosts the regeneration impact on pre-osteoblast (MC3T3-E1) cell proliferation, growth, and differentiation [94]. The impact of a hybrid nanocomposite of poly(3-hydroxybutyrate) chitosan/multiwalled carbon nanotube scaffold coated with a nanobioglass–titania scaffold on bone cell regeneration was investigated. Scanning electron microscopy (SEM) examination verified the porosity of the scaffolds in the 300–700 µm range. The incorporation of chitosan into poly(3-hydroxybutyrate) composites generates a hydrophilic environment which promotes cell motility, adhesion, and protein adsorption. Furthermore, the mixture enhances the surface roughness of the composites, stimulating bone cells to attach to the surface [108]. The different characteristics of the 3D porous collagen/functionalized multiwalled carbon nanotube/chitosan/hydroxyapatite composite were described in a bone tissue engineering application study. The XRD patterns of the lyophilized scaffolds reveal the presence of collagen, chitosan, and hydroxyapatite at 20°, 31.9°, 26.5°, 32.3°, 34.2°, 40.8°, and 75.6°. The developed scaffolds have mechanical strength of 1112 kPa. The in vitro investigation using MG-63 demonstrates reduced cytotoxicity, providing preliminary data for further research [93]. Further investigation indicated that porous chitosan gelatin with multiwalled carbon tubes functionalized with polyethylene glycol is extremely stable and might be used in bone tissue engineering applications. The drug distribution ability of the scaffolds was investigated using ciprofloxacin, which is a general antibiotic drug. According to the findings, approx. 98% of the medicines had a sustained released within 90 min [68] (Figure 4). Cancian et al. (2016) developed a novel bioactive scaffold based on a thermosensitive chitosan hydrogel. In this work, carbon nanotubes were used to stabilise the chitosan hydrogel, which offers mechanical strength and controlled release of protein therapeutics. The bioactivity of the scaffold was tested using simulated bodily fluids, and mineral formation (calcium and phosphorous) on the surface was analysed using SEM [58]. To achieve biofunctionality, a scaffold composed of carbon nanotubes and chitosan was fabricated via electrophoretic deposition. These hybrid composites show degradation as well as bone mineral formation on the surface. The in vitro biological characteristics were tested using MC3T3-E1 cells, and the findings of adherence tests demonstrate osteoblastic cell adhesion to the coated surface at various time intervals [59] (Figure 5). According to Chen et al. (2013), composites of chitosan–multiwalled carbon nanotubes with hydroxyapatite have the potential to enhance the elastic modulus and compressive strength to 1089.1 MPa and 105.5 MPa, respectively. The cell proliferation of the nanocomposites was evaluated via the CCK-8 test. The results demonstrated the best biocompatible behaviour which is suitable for bone tissue engineering applications [80].

#### Chitosan–graphene oxide nanocomposites

**Figure 6:**
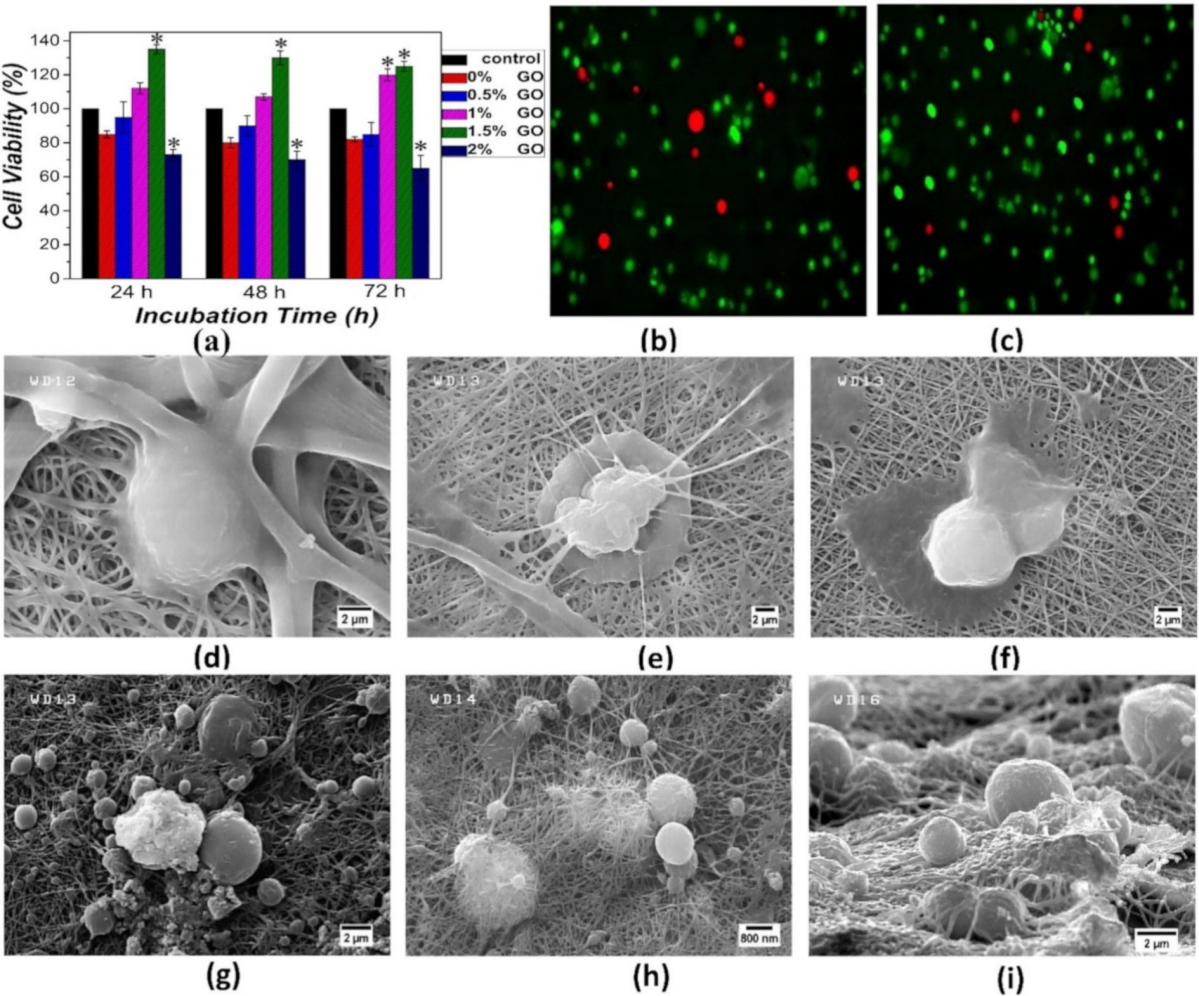
(a) The viability of mesenchymal stem cells in the developed electrospun membranes Live (green) and dead (red) staining of cells after 24 h of incubation. (b) Chitosan-based nanofibers without the addition of graphene oxide or (c) containing 1 wt % of graphene oxide. SEM images show the geometry of MSCs on the surface of membranes containing (d) 0% of graphene oxide, (e) 0.5% graphene oxide, (f) 1, (g) 1.5, and (h) 2% of graphene oxide. Figure 6 was reprinted from [72], Materials Science and Engineering: C, vol. 70, by N. Mahmoudi; A. Simchi, “On the biological performance of graphene oxide-modified chitosan/polyvinyl pyrrolidone nanocomposite membranes: In vitro and in vivo effects of graphene oxide”, pages 121–131, Copyright (2016), with permission from Elsevier. This content is not subject to CC BY 4.0.

**Figure 7:**
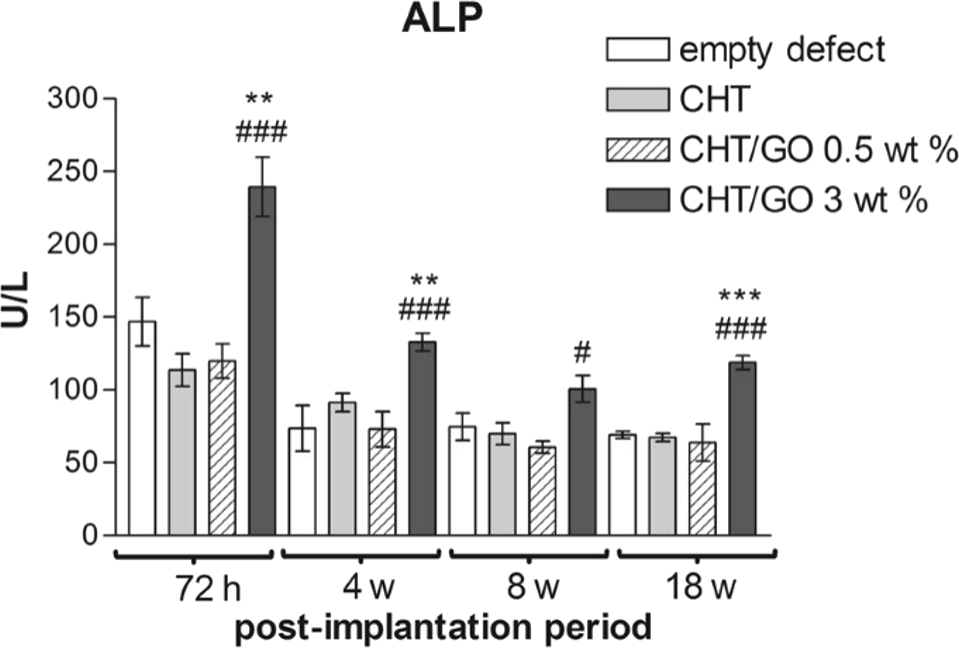
Alkaline phosphatase activity in mice calvaria defects implanted with chitosan containing graphene oxide nanomaterials after 7 h, 4 weeks, 8 weeks, and 18 weeks post-implantation. Figure 7 was reproduced from [120], (© 2017 A. Hermenean et al., published by Springer Nature, distributed under the terms of the Creative Commons Attribution 4.0 International License, https://creativecommons.org/licenses/by/4.0).

Graphene oxide is gaining much attention in biomedical applications including drug delivery, tissue engineering, and bioimaging applications due to its large surface area, antimicrobial activity, mechanical strength, osteoconductive and osteoinductive properties. In this particular study, the scaffolds of chitosan/polyvinyl alcohol/graphene oxide/hydroxyapatite/gold films were used in an orthopaedic application. The mechanical strength of the developed composites was 36.4 ± 0.7 MPa. The antibacterial activity of chitosan/polyvinyl alcohol/graphene oxide/hydroxyapatite/gold was investigated against *Escherichia coli* and *Staphylococcus aureus* with a zone of inhibition of 3–7 mm as compared to *Pseudomonas aeruginosa* and *Enterococcus faecalis*. The alkaline phosphatase staining revealed the differentiation of mesenchymal stem cells to osteoblasts [66]. The addition of polymethylmethacrylate to powder composites of chitosan/graphene oxide increased the compressive strength by 16.2%, compressive modulus by 69.1%, and bending strength by 24%. After four weeks of incubation with artificial blood plasma, hydroxyapatite bone minerals were formed. The cell survival and cell adhesion of composite-containing MG-63 cells exhibit improved biocompatibility [62]. Also, reduced graphene oxide combined with chitosan was fabricated into a hydrogel by using a tannic acid cross-linker either with acetic acid or lactic acid. The physicochemical characterisation of the composites was evaluated to prove that the composites were suitable for bone tissue engineering applications. The mechanical strength of the composites was proven through increased Young’s modulus. The surface wettability of the chitosan/reduced graphene oxide composites with specific acetic acid and lactic acid shows water contact angles of (75.40° ± 4.32°) and (36.71° ± 4.53°) [60]. The anticancer agent cisplatin was loaded into graphene oxide/hydroxyapatite/chitosan composites to enable proliferation of osteoblasts and inhibition of the development of osteosarcoma cancer cells in the work by Sumathra et al. (2018). The in vitro experiment was carried out by using the osteosarcoma MG-63 cell line. The MTT assay for the composites showed cell expansion and growth. The anticancer activity of cisplatin-loaded graphene oxide/hydroxyapatite/chitosan composites was verified by an MTT assay using A549 cells. The results revealed that the cell viability of A549 cells exceeded 23%, showing that the composites slowed osteosarcoma progression [71]. Graphene oxide-modified chitosan/polyvinylpyrrolidone developed nanofibrous structures imitating the native extracellular matrix. The potential use of this membrane for tissue engineering applications was demonstrated by using rat bone marrow mesenchymal stem cells. The cell viability of the chitosan scaffolds with 0, 0.5, 1, 1.5, and 2% of graphene oxide content was evaluated by an MTT assay. The results show that chitosan with 2% of graphene oxide has the highest cell viability. The acridine orange–propidium iodide staining was carried out after 24 h of incubation with developed nanofibers: live cells were stained in green and dead cells were stained in red, which were identified by fluorescence imaging. Cells adhered on nanofibers show a better cell phenotype, and this was corroborated by morphological characterisation via SEM [72] (Figure 6). Misra and colleagues developed chitosan–graphene nanocomposite scaffolds that modify cell–scaffold interactions. Through cell viability, porosity measurements, in vitro degradation, and degradation tests, researchers determined that the composites showed increased biocompatibility and promoted cell proliferation and growth, in addition to having a steady degradation rate [61]. In addition, a layer-by-layer assembly approach has been used to create a graphene oxide with chitosan and hydroxyapatite nanocomposite film for a possible use in bone tissue regeneration. As per study results, the combined chitosan and hydroxyapatite nanocomposite film provides an excellent substrate for the growth of mouse mesenchymal stem cells. Due to the porosity nature of the scaffolds, the anti-inflammatory drug aspirin was entrapped and gradually released [73]. Hermenean et al. (2017) reported that chitosan–graphene oxide scaffolds were used for treating critical-size mouse calvarial defects. Scaffolds of chitosan with 0, 0.5, and 3 wt % of graphene oxide were implanted on four-week-old CD1 mice into calvarial bone defects. The alkaline phosphatase activity was measured from the blood samples of the animal and the results show that the alkaline phosphatase activity was higher on the chitosan with a combination of silver, gold, copper, and 3 wt % of graphene oxide after 7 h, 4 weeks, 8 weeks, and 18 weeks post-implantation (Figure 7). The histological findings show that new bone formation in the chitosan scaffolds containing graphene oxide was higher at 18 weeks [120].

### Chitosan with biosilica for bone tissue engineering

Silica-based biomaterials are widely used in bone tissue engineering due to their superior biocompatibility and cell proliferation ability. In addition, biodegradability and higher mechanical strength make them more versatile to be used as a synthetic bone implant. In addition, silica-based tailored materials such as mesoporous bioactive glass possess exceptional osteogenic properties [121–122]. Diatomite and chitosan-containing scaffolding systems were developed by Tamburaci and co-workers. The developed composite shows excellent cell proliferation, mineralisation, and alkaline phosphatase activity on MG-63 cells, Saos-2 cells, and human osteoblasts, demonstrating its significance in bone tissue engineering [54]. Lemos and colleagues synthesized nanocomposite films comprising chitosan and bioactive glass, as well as a hybrid composition of chitosan and bioactive glass. The chitosan with 20% of bioactive glass has a mechanical tensile strength of 67 MPa. In addition, excellent biomineralisation was observed in SBF which shows the ability of bone mineral formation of chitosan nanocomposites [64]. Chitosan and tetraethoxysilane-based aerogels show cell attachment and proliferation of human osteoblasts [63]. In another study, Li et al. (2015) used the electrospinning method to fabricate chitosan and MSN-containing nanofibers. In addition, an increase in mechanical strength was observed with an increase in the MSN content. Further in vitro assays reveal that nanofibres slowly degrade and have a high swelling ratio. Besides, in vitro biological assays with MC3T3-E1 cells show that nanofibres promote cell proliferation, alkaline phosphatase activity, and induce mineralisation [65].

Chitosan and biosilica-containing nanocomposites which include chitosan/ octa(tetramethylammonium)polyhedral silsesquioxane, chitosan–nanoSiO_2_–chondroitin sulphate, chitosan–nanoSiO_2_–gelatin, and chitosan–bioglass/hydroxyapatite/halloysite nanotubes have remarkable osteogenic characteristics [82–84,123]. Chitosan and silica-based microspheres were produced by using sol–gel followed by emulsification and cross-linking methods. Next, vancomycin hydrochloride was encapsulated into the microspheres. In vitro biomineralisation tests show apatite formation on the surface of the microspheres. In addition, a sustainable drug release profile was detected. This finding reveals that the produced microspheres have promising applications in bone tissue engineering [55].

### 3D printing chitosan material for bone tissue engineering

The 3D printing is an emerging technique used in tissue engineering, in which biomaterials are 3D printed to mimic the native tissue architecture. In bone tissue engineering and regenerative medicine, the 3D scaffold system was used to imitate bone tissue anatomy. These scaffold systems consist of composite scaffolds of polymeric materials. Among other composite materials, chitosan composites were widely used in bone tissue engineering applications due to their porous nature and biocompatibility with osteoblast stem cells. Chitosan with hydroxyapatite were used to 3D print the scaffolds used to improve the mechanical strength of the bone implant. These dense and cylindrical structures of Ø 10 × 10 mm were 3D printed and 40 wt % lactic acid was used as the binder solution. The results show that the compressive strength of the 3D-printed samples was 16.32 MPa, with a porosity of 37.1% [124]. Nazeer et al. used 3D-printed poly (lactic acid) with chitosan and hydroxyapatite scaffolds for bone repair applications. The honeycomb and rectilinear pattern of the scaffolds were printed through a fused deposition model at 210 °C with a layer height of 200 µm. The in vitro ability of the 3D-printed scaffolds was evaluated in human osteosarcoma cells and the results show that the composites are biocompatible and nontoxic to the cells [125]. An extrusion-based 3D printing of methacrylate chitosan–laponite nanosilicate composites was used for bone tissue engineering applications. The MC3T3-E1 osteoblasts cultured on 3D-printed scaffolds show increased cell viability, cell growth, and bone mineral formation. The SEM analysis results show that osteoblasts seeded onto methacrylate chitosan–laponite nanosilicate composites show similar extracellular growth [126]. Shafiei et al. (2019) developed egg-shell-based 3D-printed multiphasic calcium phosphate scaffolds to induce an osteoinductive character for bone tissue repair. The 3D-printed scaffolds achieved an interconnected porosity of ≈60.7% which promoted adhesion and migration of mesenchymal stem cells confirmed by cell adhesion and morphology studies. The cell differentiation activity of 3D-printed scaffolds was confirmed by the alkaline phosphatase activity assay on the 14th day [127]. Liu et al. (2020) has used 3D printing and electrospinning for the fabrication of scaffolds for cartilage tissue engineering applications. The 2 cm × 2 cm PLGA electrospun nanofibers were prepared by electrospinning which incorporated those with hydroxybutyl chitosan hydrogels. The polycaprolactone scaffold was 3D printed and reinforced with hydrogel scaffolds to mimic the internal structure of cartilage. Human mesenchymal stem cell differentiation in 3D-printed scaffolds showed the differentiation ability of the cartilage tissue [128].

### Future approaches

Nanomaterials are widely used in the fabrication of scaffolds as they significantly mimic the extracellular matrix and rapidly generate and stimulate functional bone tissue in defective areas. In addition, nanomaterial-based grafts are biocompatible and nontoxic and can more effectively promote osteoconductivity, osteoinductivity, and neovascularization. Also, due to a higher surface area, nanomaterials can promote wettability and protein adsorption which can facilitate osteogenesis [129]. Biomaterials such as chitosan and its composites containing bioactive metals draw much attention in tissue engineering and regenerative applications. Chitosan-based composites are now being studied in wound healing, bone and cartilage regeneration, and other applications. Furthermore, chitosan-containing polymer composites are being extensively explored for drug delivery in targeted tumour treatment and nucleic acid delivery in genetic engineering applications. More research is required to optimise chitosan composites utilised in scaffolds in order to achieve vascularization and rapid tissue growth. To achieve tissue scaffold maturation, research into the development of scaffolds with supplied growth factors, programmable degradation rate, and good mechanical stiffness with improved bioactivity is required. Furthermore, mathematical modelling and clinical imaging of the scaffolds will assist in the establishment of its micro- and nanoarchitecture, which will aid in the regulation and activation of the immune system for bone tissue repair and regeneration.

## Conclusion

Chitosan is a naturally occurring biopolymer with appropriate mechanical characteristics. Nanocomposites formed by chitosan and metals, such as silver, gold, copper, titanium oxide, and zinc oxide were studied in the treatment of bone tissue defects and have proven to be effective in bone tissue repairing processes. Also, chitosan combined with carbon nanomaterials, graphene oxide, and biosilica nanocomposites has bioconductive and osteoinductive behaviour with suitable mechanical strength. The chitosan–graphene oxide composite is gaining much attention in biomedical applications including drug delivery, tissue engineering, and bioimaging, due to its large surface area, antimicrobial activity, mechanical strength, and osteoconductive and osteoinductive properties. The 3D bioprinting technologies help to mimic micro- and nanoarchitectures of the bone by printing cells alongside developed bioinks which maintain the scaffolds in a mature stage. Following this, treating bone calvarial defects with a natural, mechanical, and biochemical environment with accomplished osteoblast differentiation and proliferation has been addressed by using chitosan–graphene nanocomposite materials.
